# The association between social determinants of health and psychological distress during the COVID-19 pandemic: a secondary analysis among four racial/ethnic groups

**DOI:** 10.1186/s12889-022-14486-x

**Published:** 2022-11-28

**Authors:** Yan Luo, Qingyi Li, Haelim Jeong, Leah Cheatham

**Affiliations:** 1 School of Social Work, The University of Alabama, Tuscaloosa, AL 35401 USA; 2 The University of Hawaiʻi at Mānoa, HI Honolulu, USA; 3 Department of Psychology, Cornell University, Ithaca, NY 14850 USA

**Keywords:** Social determinants of health, Racial/ethnic disparities, Psychological distress, Food insecurity, Neighborhood safety, Delayed medical care

## Abstract

**Background:**

Racial disparities in psychological distress associated with COVID-19 remain unclear in the U.S. This study aims to investigate the associations between social determinants of health and COVID-19-related psychological distress across different racial/ethnic groups in the US (i.e., non-Hispanic Whites, Hispanic, non-Hispanic Asians, and non-Hispanic African Americans).

**Methods:**

This study used cross-sectional data from the 2020 California Health Interview Survey Adult Data Files (*N* = 21,280). Adjusting for covariates—including age, gender, COVID-19 pandemic challenges, and risk of severe illness from COVID-19—four sets of weighted binary logistic regressions were conducted.

**Results:**

The rates of moderate/severe psychological distress significantly varied across four racial/ethnic groups (*p* < 0.001), with the highest rate found in the Hispanic group. Across the five domains of social determinants of health, we found that unemployment, food insecurity, housing instability, high educational attainment, usual source of health care, delayed medical care, and low neighborhood social cohesion and safety were associated with high levels of psychological distress in at least one racial/ethnic group (*p* < 0.05).

**Conclusion:**

Our study suggests that Hispanic adults face more adverse social determinants of health and are disproportionately impacted by the pandemic. Public health practice and policy should highlight social determinants of heath that are associated with different racial/ethnic groups and develop tailored programs to reduce psychological distress.

## Introduction

### Psychological distress in the COVID-19 pandemic

In March 2020, the World Health Organization declared the outbreak of COVID-19 a pandemic, indicating the outbreak had become an acute global public health concern. The emergence of the COVID-19 pandemic, followed by city lockdowns, closures of schools and social services, social isolation, and economic recessions, may result in significant and long-term psychological distress across different populations. Petzold et al. (2020) reported that approximately 4 hours per day were spent thinking about the COVID-19 pandemic among 6,509 general respondents in Germany. This excessive amount of time spent thinking about COVID-19, to some extent, made individuals vulnerable to psychological distress in response to the pandemic [1].

Researchers and scholars from different cultures examined predictors of psychological distress during the COVID-19 crisis. A recent systematic review synthesized existing research addressing mental health well-being in the general population across eight countries (i.e., U.S., China, Italy, Turkey, Spain, Denmark, Iran, and Nepal) and summarized risk factors associated with psychological distress [2]. Xiong and colleagues (2020) presented that chronic/psychiatric illness, unemployment, and frequent exposure to social media or news related to the COVID-19 pandemic were associated with increased levels of psychological distress among the general population. Another recent study explored the relationship between the COVID-19 pandemic and psychological distress among three Chinese population groups: COVID-19 patients, individuals under quarantine, and the general public [3]. Zhang et al. (2020) found that COVID-19 patients and the general public showed increased severe depressive symptoms and anxiety-related behaviors compared to individuals under quarantine [3]. Variation among particular groups’ psychological distress suggests the importance of examining the psychological effects of the pandemic in nuanced ways, including attention toward variation by demographic characteristics.

A growing body of research exploring sociodemographic factors associated with psychological distress indicated that the COVID-19 pandemic impacted psychological distress most dramatically among young adults and those of female gender [2, 4–7]. However, few studies have examined the psychosocial risk factors of psychological distress in the COVID-19 public health crisis across cultures within the U.S. A dearth of research has focused on risk factors of racial/ethnic disparities in psychological distress in response to the pandemic. Given the cultural diversity within the US, including citizens of different races and ethnicities [8], attention toward the unique impact of the pandemic within the US for specific groups of people will be critical to fully understanding the psychological impact of the COVID-19 pandemic.

### Racial disparities in the COVID-19 pandemic

Racial disparities in psychological distress associated with COVID-19 remain unclear in the US. Chowkwanyun et al. (2020) proposed that historically marginalized populations will be affected by the COVID-19 disproportionately regarding the experience of epidemics [9]. A handful of studies have illustrated the racial/ethnic disparities of COVID-19 testing, infection and mortality rates, and hospitalization rates during the pandemic. Anyane-Yeboa et al. (2020) assessed the rates of COVID-19 infections and death across different racial/ethnic groups in sixteen states in the US [10]. Findings suggested that African Americans and Hispanics experienced higher rates of COVID-19 infection than the general White population; Additionally, African Americans experienced the highest COVID-19 death rate [10]. Several later studies showed similar results: that African American populations demonstrated the highest mortality rates of COVID-19 among all U.S. racial/ethnic groups [11–13]. Renelus et al. (2021) found that African Americans were two times more likely than Whites to require COVID-19 hospitalization, and Hispanics were more likely to suffer in-hospital mortality from COVID-19 compared to Whites during the height of the pandemic in New York City. The COVID-19 pandemic is unique in both its severity and scope of physical, social, and economic consequences.

### Psychological distress in COVID-19 under social determinants of health perspectives

The social determinants of health (SDOH) framework focuses on non-medical factors that influence health and mental health conditions. The SDOH framework is responsible for explaining health inequalities and disparities across population groups. Research has demonstrated the ways in which social determinants have exacerbated health disparities during the COVID-19 pandemic [14]. To address a wide range of health outcomes and risks, the SDOH framework can be categorized into five groups [15], including 1) economic stability (e.g., employment, income, medical bills, food security), 2) education access and quality (e.g., literacy, early childhood education, language), 3) health care access and quality (e.g., health coverage, provider availability, quality of care), 4) neighborhood and built environment (e.g., transportation, parks, walkability), and 5) social and community context (e.g., social integration, support system, stress). A large body of research investigated psychological distress applying the SDOH framework across different countries and cultures [16–20]. However, little research has focused on social determinants of psychological distress across different racial/ethnic groups in the US during the unprecedented COVID-19 pandemic.

### The present study

Guided by SDOH framework, we aim to 1) assess the levels of psychological distress across four racial/ethnic groups (i.e., non-Hispanic Whites, Hispanics, non-Hispanic Asians, and non-Hispanic African Americans), and 2) investigate the associations between SDOH and psychological distress across racial/ethnic groups.

## Method

### Data source

The present study used cross-sectional data from the 2020 California Health Interview Survey (CHIS) Adult Data Files. From March 9, 2020 to October 31, 2020, the data were collected through a population-based multimode (web and telephone) survey designed to examine public health and health care issues among residential and non-institutionalized populations in California [21, 22]. By using a stratified address-based sample (ABS) methodology, CHIS provided estimates for adults in most counties and groups of counties with small populations, as well as California’s overall population, including major racial/ethnic groups and several smaller racial/ethnic subgroups [21]. Selection of specific targeted groups of interest for oversampling was achieved through statistical modeling that was built by using CHIS 2017–2018. Samples were selected through a two-stage sample design. In the first stage of sampling, households were selected through stratified ABS. In the second stage, households were contacted and one adult resident of the household (18 years of age or older) was randomly chosen to be interviewed. Respondents received an initial invitation letter with $2.00 pre-incentive, followed by a reminder postcard, and a standard letter and final postcard for all non-respondents [21]. Participation in the study is voluntary as participants can choose whether to participate after receiving the invitation. More details on sampling and data collection are reported in CHIS 2020 Methodology Report Series [21]. The completion rate was 11.4% [22]. Missing values were replaced through imputation methods [21]. The CHIS public-use dataset was approved for use without seeking institutional review board approval by the first author’s institution.

### Participants

Participants were divided into four race/ethnicity groups: non-Hispanic Whites (63.32%), Hispanics (20.29%), non-Hispanic Asians (12.90%), and non-Hispanic African Americans (3.50%). The total analytic sample size is 21,280. Participants were evenly distributed in three different age groups: young adults between 18–44 years old (29.17%), middle-aged adults between 45–64 years old (35.49%), and older adults over 65 years old (35.33%). Slightly more than half of participants were female (56.33%).

### Measures

#### Outcome variable

The outcome variable in this study was psychological distress, which was measured by The Kessler Psychological Distress Scale (K6) [23]. Participants were asked “during the past 30 days, about how often did you feel: (1) nervous; (2) hopeless; (3) restless or fidgety; (4) so depressed that nothing could cheer you up; (5) that everything was an effort; (6) worthless (1 = all of the time, 2 = most of the time, 3 = some of the time, 4 a little of the time, 5 = none of the time”; Cronbach’s alpha = 0.87). The responses were reverse-coded (i.e., 5 = all of the time…1 = none of the time) and summed across the six questions to create the total psychological distress score (range: 6–30), in which higher score indicated higher level of psychological distress. The outcome variable was obtained by categorizing the total psychological distress score into two groups, where scores ranging between 6–10 represented low psychological distress while scores equal to or higher than 11 represented moderate/severe psychological distress [23]. Compared to no/low psychological distress, moderate/severe psychological distress impacts daily functioning and warrants clinical attention [23].

#### Independent variables: SDOH

Independent variables were framed according to the SDOH model [15]. Five domains were identified: economic stability, education, social and community context, health care access and quality, and neighborhood and built environment.

In terms of economic stability, variables capturing employment, food security, and home ownership were included in this study. Participants were asked “Which of the following were you doing last week: working at a job or business, with a job or business but not at work, looking for work, and not working at a job or business?” Working at a job/business and with a job/business but not at work were collapsed into one category “1 = employed”, while looking for work and not working at a job/business were collapsed into the other category “0 = unemployed”. Regarding food security, only participants whose total annual household income was below 200% federal poverty level (FPL) were asked questions regarding food security (i.e., “In the past year, how often food did not last and could not afford more, how often could not afford to eat balanced meals, did anyone in the household ever cut/skip meals for money and how often that happened, did participants eat less than they should because of money, were participants ever hungry but did not eat because of money?”). CHIS summarized those questions into one variable representing food security status (1 = food security, 2 = food insecurity without hunger, 3 = food insecurity with hunger). In the present study, poverty and food security were combined into one variable: 1 = food insecurity, 2 = food security but under 200% FPL, 3 = above 200% FPL. Home ownership was measured by asking participants “do you own or rent your home?” (0 = other arrangement/rent, 1 = own). Home ownership, employment, and food security were analyzed as categorical variables.

Educational attainment and English proficiency were included and also analyzed as categorical variables. Educational attainment of participants was dichotomized into “0 = below bachelor’s degree” and “1 = bachelor’s degree or above”. The cutoff was based on previous studies that investigated association between education and physical/mental health [24, 25]. In terms of English proficiency, participants were asked “what languages do you speak at home?” Participants who indicated speaking a language other than English at home were asked the following question: “Would you say you speak English very well/well/not well/not at all?” A final variable of English proficiency was constructed by aggregating responses of these two questions into one categorical variable (1 = not well/not at all, 2 = well/very well, 3 = only speak English).

Regarding social and community context, neighborhood social cohesion was included and analyzed as a continuous variable. To measure social cohesion, CHIS asked participants how much they agree or disagree (indicated by a four-point Likert scale where 1 = strongly disagree and 4 = strongly agree) with the following three items: (1) people in my neighborhood are willing to help each other; (2) people in this neighborhood generally do not get along with each other; (3) people in this neighborhood can be trusted. The second item was reverse coded, and the final variable was constructed by summing the responses for three items. Higher scores indicated higher levels of neighborhood social cohesion (range = 3–12).

For health care access and quality, having a usual source (except emergency room) to go for health care, and delayed medical care were included and analyzed as categorical variables. Participants were asked “Is there a place that you usually go to when you are sick or need advice about your health?” (yes/no) and a following question was asked to participants who said “yes”: “What kind of place do you go to most often—a medical doctor's office, a clinic or hospital clinic, an emergency room, or some other place?” (1 = medical doctor’s office, 2 = clinic/hospital clinic, 3 = emergency room, 4 = other place). The final variable of having a usual source (except emergency room) to go for health care was constructed by combining the above two questions (0 = no, 1 = yes). Delayed medical care was measured by asking “During the past 12 months, did you delay or not get any other medical care you felt you needed?” (0 = no, 1 = yes).

Lastly, with regard to neighborhood and built environment, neighborhood safety was included. Participants were asked “Do you feel safe in your neighborhood?” (1 = all of the time, 2 = most of the time, 3 = some of the time, 4 = none of the time). In the present study, the variable was reverse coded (1 = none of the time, 2 = some of the time, 3 = most of the time, 4 = all of the time) and analyzed as a continuous variable.

#### Covariates

Demographics, COVID-19 pandemic challenges, and risk of severe illness from COVID-19 [26] were added in the analysis as covariates. Demographics included age (1 = young adults 18–44 years old, 2 = middle-aged adults 45–64 years old, 3 = older adults 65–74 years old, 4 = older-old adults 75 years old and above) and gender (0 = male, 1 = female).

In terms of COVID-19 pandemic challenges, participants were asked “Have you experienced any of the following situations because of the Coronavirus or COVID-19 outbreak (no = 0, yes = 1)”: (1) I’ve lost my regular job; (2) I’ve had a reduction in hours, or a reduction in income; (3) I’ve switched to working from home; (4) I’ve continued to report to work because I was an essential worker; (5) I’ve had difficulty in obtaining childcare, or had an increased in childcare expenses; (6) I’ve had financial difficulties with paying rent or mortgage; (7) I’ve had financial difficulties with basic necessities, such as paying bills, tuition, affording groceries, etc.; (8) I’ve been treated unfairly because of my race/ethnicity; (9) I’ve been experiencing increased mental health challenges; (10) I’ve been experiencing other challenges. The total score of COVID-19 pandemic challenges was obtained by summing responses to all above 10 items to create a score, which was analyzed as a continuous variable (range = 0–10).

Those who indicated having asthma, diabetes, high blood pressure, heart disease, obesity, or smoking 100 or more cigarettes in their entire lifetime were identified as having higher likelihood of getting severely ill from COVID-19 [26]. Those conditions were summed to represent risk of severe illness from COVID-19 and the final variable was analyzed as a continuous variable (range = 0–6).

### Statistical analysis

Descriptive analysis was conducted for all variables and each item of the outcome measure, the K6 score. Bivariate tests were conducted to explore whether participants were significantly different in each category of SDOH and covariates across four race/ethnicity groups (Pearson chi-square test for categorical variable and F-test for continuous variables with using non-Hispanic Whites as the reference group). Unadjusted relationship between race/ethnicity and psychological distress level as well as specific items was also explored with bivariate analysis (F-test with using non-Hispanic Whites as the reference group). Lastly, adjusting for covariates—including age, gender, COVID-19 pandemic challenges, and risk of severe illness from COVID-19—four sets of weighted binary logistic regressions were conducted to examine the relationship between SDOH and psychological distress across four racial/ethnic groups. The final sample weight was used to obtain population estimates and 80 jackknife replicate weights were used to obtain variation estimates. The data analysis was conducted using Statistics and Data/Special Edition 15.1 (Stata/SE 15.1), which allowed for incorporation of jackknife replicate weights to assess variation estimation.

## Results

### Descriptive information of SDOH and covariates

Table 1 reports the descriptive information of five domains of SDOH (i.e., economic stability, education, social and community context, health care access and quality, and neighborhood and built environment), and covariates among participants. Participants reported a moderate level of neighborhood social cohesion of their community (*M* = 7.97, *SD* = 0.97, range = 3 to 12). For neighborhood and built environment, participants reported a relatively high level of neighborhood safety (*M* = *3.38, SD* = *0.63*, range = 1 to 4). The mean number of COVID-19 pandemic challenges faced by participants was 0.79 (*SD* = *0.92*, range = 0 to 8) and participants reported an average of 1.60 conditions that put them at risk of severe illness form COVID-19 (*SD* = *1.21*, range = 0 to 6).

**Table 1:** Descriptive information (*N* = 21,280 ^a^)

		All (*N* = 21,280)	Non-Hispanic Whites (*n* = 13,474)	Hispanics (*n* = 4317)	Non-Hispanic Asians (*n* = 2745)	Non-Hispanic African Americans (*n* = 744)
		N (%) or Mean (SD) ^b^	Weighted % Or Mean ^b^			
**Social Determinants of Health**						
***Economic stability***						
Employment	Unemployed	9248 (42.13)	39.83	32.04	33.44	38.87
	Employed	12,701 (57.87)	60.17	67.96	66.56	61.13
Food security	Food insecurity	1352 (6.16)	4.81	16.45	6.89	14.42
	Food security but under 200% FPL	2836 (12.92)	11.05	27.57	17.48	14.18
	Above 200% FPL	17,761 (80.92)	84.14	55.98	75.63	71.4
Housing instability	Other arrangement or rent	6126 (28.54)	34.30	53.21	42.51	57.98
	Own	15,339 (71.46)	65.70	49.79	57.49	42.02
***Education***						
Educational attainment	Below bachelor’s degree	9436 (42.99)	48.67	75.7	39.49	59.64
	Bachelor’s degree or above	12,513 (57.01)	51.33	24.3	60.51	40.36
English proficiency	Not well/not at all	797 (3.63)	0.56	16.80	17.23	0
	Well/very well	5084 (23.16)	10.23	59.40	56.83	7.32
	Only speak English	16,068 (73.21)	89.21	23.80	25.94	92.68
***Social and community context***						
Neighborhood social cohesion (Range = 3–12)		7.97 (0.97)	7.94	7.73***	7.90	7.60***
***Health care access and quality***						
Usual source of health care (except Emergency care)	No	2153 (9.81)	10.88	20.13	15.12	12.67
	Yes	19,796 (90.19)	89.12	79.87	84.88	87.33
Delayed medical care	No	17,988 (81.95)	81.12	85.29	84.44	86.85
	Yes	3961 (18.05)	18.88	14.71	15.56	13.15
***Neighborhood and built environment***						
Neighborhood safety (Range = 1–4)		3.38 (0.63)	3.39	3.17***	3.18***	3.18***
**Covariates**						
Age	Young adults (18–44 yrs)	6462 (29.44)	36.59	58.16	49.08	35.5
	Middle-aged adults (45–64 yrs)	7804 (35.56)	31.75	30.07	33.85	41.26
	Older adults (65yrs and older)	7683 (35.00)	31.65	11.77	17.08	23.24
Gender	Male	9575 (43.62)	49.4	49.63	47.38	47.10
	Female	12,374 (56.38)	50.6	50.37	52.62	52.90
COVID-19 pandemic challenge (Range = 0–8)		0.79 (0.92)	0.80	1.02***	0.97***	1.09***
Risk of severe illness from COVID-19 (Range = 0–6)		1.60 (1.21)	1.58	1.44***	1.10***	1.84***

^a^ The total sample size of the study may not be the same as the total sample size of the survey due to missing values

^b^ N (%) for categorical variables and Mean (SD) for continuous variables

^*^ *p* < 0.05

^**^ *p* < 0.01

^***^ *p* < 0.001

### Levels of psychological distress across race/ethnicity

Table 2 shows psychological distress levels among participants in different racial/ethnic groups. Around one-third of participants reported moderate/severe psychological distress during the past 30 days. The percentage of participants with moderate/severe psychological distress significantly varied across four racial/ethnic groups (non-Hispanic Whites [NHWs]: 36.95%, Hispanic: 40.58%, non-Hispanic Asians [NHAs]: 39.92%, non-Hispanic African-Americans [NHAA]: 30.98%, *p* < 0.001). The overall psychological distress level among participants in the study was around 10 (*M* = 9.94, *SD* = 4.02, range = 6 to 30).

**Table 2:** Psychological distress across race/ethnicity (*N* = 21,280)

	All	Non-Hispanic Whites	Hispanics	Non-Hispanic Asians	Non-Hispanic African Americans	
During the past 30 days	N (%)	Weighted %				*p*-Value ^a^
Moderate or severe psychological distress	7359 (33.54)	36.95	40.58	39.92	30.98	0.001***
	Mean (SD)	Weighted Mean ^b^				
Psychological distress score (range = 6–30)	9.94 (4.02)	10.24	10.77***	10.47	9.64**	
Feel nervous (range = 1–5)	2.04 (0.94)	2.08	2.19***	2.08	1.93***	
Feel hopeless (range = 1–5))	1.50 (0.82)	1.55	1.65***	1.62*	1.45*	
Feel restless (range = 1–5))	1.98 (0.97)	2.07	2.12	1.97***	1.90***	
Feel depressed (range = 1–5)	1.37 (0.73)	1.38	1.52***	1.49***	1.38	
Feel everything is an effort (range = 1–5)	1.74 (0.97)	1.80	1.85***	1.85***	1.72**	
Feel worthless (range = 1–5)	1.31 (0.72)	1.35	1.44	1.46	1.26	

^a^ *p*-Value for Chi-square test

^b^ *p*-Value for F-test using non-Hispanic Whites as reference group

^*^p < 0.05

^**^p < 0.01

^***^p < 0.001

### Associations between SDOH and psychological distress

Table 3 shows the associations between SDOH and psychological distress among participants in four racial/ethnic groups, controlling for age, gender, pandemic challenges, and severe illness risks from COVID-19. Being employed was significantly associated with lower odds of moderate/severe psychological distress among NHWs (*OR* = 0.71, *95% CI* = 0.59—0.84), Hispanics (*OR* = 0.65, *95% CI* = 0.52—0.80), and NHAAs (*OR* = 0.47, *95% CI* = 0.28—0.77). The effect of employment was strongest among NHAAs compared to other groups. Food security was negatively associated with psychological distress among NHWs and Hispanics. Among those two groups, participants who were food secure but living under 200% FPL had lower odds of moderate/severe psychological distress compared to participants with food insecurity: NHWs (*OR* = 0.57, *95% CI* = 0.41—0.78), Hispanics (*OR* = 0.58, *95% CI* = 0.44—0.78). Similar relationships were found for participants above 200% FPL among NHW and Hispanic groups—both reported lower levels of psychological distress compared to those who reported food insecurity: NHWs (*OR* = 0.47, *95% CI* = 0.34—0.64), Hispanics (*OR* = 0.65, *95% CI* = 0.49—0.85). Home ownership was significantly associated with psychological distress only among NHWs: NHWs who owned a home had a lower likelihood having moderate/severe psychological distress compared to those who rented a home or had other arrangements (*OR* = 0.76, *95% CI* = 0.66—0.88).

**Table 3:** Weighted binary logistic regression on social determinants of health predicting psychological distress across race/ethnicity ^a^

	Non-Hispanic Whites	Hispanics	Non-Hispanic Asians	Non-Hispanic African Americans
	OR (95% CI)	OR (95% CI)	OR (95% CI)	OR (95% CI)
**Social Determinants of Health**				
***Economic stability***				
Employment (ref = unemployed)				
Employed	0.71*** **(0.59, 0.84)**	0.65*** **(0.52, 0.80)**	0.88 (0.65, 1.19)	0.47** **(0.28, 0.77)**
Food security (ref = Food insecurity)				
Food security but below 200% FPL	0.57** **(0.41, 0.78)**	0.58*** **(0.44, 0.78)**	0.72 (0.40, 1.30)	0.76 (0.30, 1.88)
Above 200% FPL	0.47*** **(0.34, 0.64)**	0.65** **(0.49, 0.85)**	0.73 (0.43, 1.22)	0.90 (0.47, 1.72)
Housing stability (ref = rent or other arrangement)				
Owning a home	0.76*** **(0.66, 0.88)**	0.97 (0.81, 1.17)	0.85 (0.64, 1.14)	1.23 (0.74, 2.03)
***Education***				
Educational Attainment (ref = below bachelor’s degree)				
Bachelor’s degree or above	1.05 (0.94, 1.17)	1.12 (0.90, 1.38)	0.98 (0.71, 1.34)	1.72** **(1.17, 2.54)**
English Proficiency (ref = not well/not at all) ^b^				
Speak English well/very well	0.17 (0.02, 1.42)	1.01 (0.74, 1.38)	0.70 (0.45, 1.11)	
Only speak English	0.20 (0.02, 1.74)	1.13 (0.82, 1.57)	0.66 (0.43, 1.00)	1.71 (0.57, 5.10)
***Health care access and quality***				
Usual source for health care (except EMR) (ref = no)				
Yes	0.81 (0.66, 1.00)	0.84 (0.67, 1.06)	0.79 (0.55, 1.13)	0.43* **(0.22, 0.83)**
Delayed Medical Care (ref = No)				
Yes	2.64*** **(2.30, 3.04)**	2.40*** **(1.96, 2.95)**	2.25*** **(1.60, 3.18)**	3.10*** **(1.73, 5.57)**
***Social and community context***				
Neighborhood social cohesion	0.91* **(0.85, 0.99)**	0.93 (0.85, 1.02)	1.00 (0.86, 1.15)	0.97 (0.78, 1.22)
***Neighborhood and built environment***				
Neighborhood safety	0.69*** **(0.62, 0.76)**	0.69*** **(0.61, 0.78)**	0.69*** **(0.59, 0.82)**	0.56** **(0.39, 0.80)**
**Covariates**				
Age (ref = young adults 18-44yrs)				
Middle-aged adults (45–64 yrs)	0.44*** **(0.37, 0.53)**	0.50*** **(0.41, 0.61)**	0.36*** **(0.28, 0.46)**	0.36*** **(0.21, 0.59)**
Older adults (65yrs and older)	0.23*** **(0.19, 0.29)**	0.34*** **(0.25, 0.46)**	0.31*** **(0.19, 0.51)**	0.22*** **(0.11, 0.43)**
Gender (ref = male)				
Female	1.35*** **(1.18, 1.55)**	1.25* **(1.03, 1.52)**	1.27* **(1.01, 1.59)**	1.53 (1.00, 2.32)
COVID-19 pandemic challenge	1.32*** **(1.20, 1.45)**	1.27*** **(1.15, 1.39)**	1.46*** **(1.27, 1.68)**	1.33* **(1.02, 1.74)**
Risk of severe illness from COVID-19	1.13*** **(1.07, 1.20)**	1.10* **(1.01, 1.20)**	1.08 (0.96, 1.21)	1.40** **(1.14, 1.71)**
Number of observations	13,275	4160	2644	718
Goodness of fit test *p-*value ^c^	0.36	0.09	0.64	0.48

^a^ The total sample size of the study may not be the same as the total sample size of the survey due to missing values

^b^ Speak English well/very well was used as reference group for non-Hispanic African Americans

^c^ Non-significant *p*-values indicate good fit

^*^p < 0.05

^**^p < 0.01

^***^p < 0.001

Educational attainment was only significantly linked to psychological distress levels among NHAA respondents, indicating NHAAs with a bachelor’s degree or higher degree were more likely to have moderate/severe psychological distress (*OR* = 1.72, *95% CI* = 1.17 – 2.54). Among variables in the health care access and quality domain, experiencing delayed medical care was positively associated with the likelihood of reporting moderate/severe psychological distress among all groups: NHWs (*OR* = 2.64, *95% CI* = 2.30 – 3.04), Hispanics (*OR* = 2.40, *95% CI* = 1.96 – 2.95), NHAs (*OR* = 2.25, *95% CI* = 1.60 – 3.18), and NHAAs (*OR* = 3.10, *95% CI* = 1.73 – 5.57). The effect of experiencing delayed medical care was the strongest among NHAAs.

Participants with higher levels of neighborhood social cohesion appeared to have slightly lower odds of having moderate/severe psychological distress among NHWs (*OR* = 0.91, *95% CI* = 0.85 – 0.99). Higher levels of self-reported neighborhood safety were associated with a lower likelihood of reporting moderate/severe psychological distress among all groups: NHWs (*OR* = 0.69, *95% CI* = 0.62 – 0.76), Hispanics (*OR* = 0.69, *95% CI* = 0.61 – 0.78), NHAs (*OR* = 0.69, *95% CI* = 0.59 – 0.82), and NHAAs (*OR* = 0.56, *95% CI* = 0.39 – 0.80). The effect of neighborhood safety was strongest among NHAAs.

## Discussion

### Racial/ethnic disparity in psychological distress

We found that the rates of moderate/severe psychological distress among participants were significantly different across four racial/ethnic groups. The Hispanic group reported the highest rate of moderate/severe psychological distress, which is in line with recent studies [27, 28]. This study also showed that Hispanics had the highest rate of food insecurity while living under 200% FPL, the lowest rate of English proficiency, the lowest rate of reporting a usual source of health care, as well as the lowest rate of neighborhood safety. Our study indicates that the Hispanic group was disproportionately impacted by the pandemic, which contributes to their relatively poor mental health outcomes.

In our study, NHAAs had the lowest rate of moderate/severe psychological distress among four groups, which is consistent with previous studies. For instance, recent studies found that NHAA adults were less likely to report depressive symptoms or anxiety compared to other racial/ethnic groups [29–31]. One plausible explanation is that the mental health advantage of African Americans might be related to high religious involvement or this group’s higher standard for being mentally stressed [29]. However, our study also found African Americans showed the highest housing instability and risks of severe illness from COVID-19. Another study from Riehm and colleagues (2021) reported that NHAAs were more likely to have high resilience during the pandemic despite facing more stressors [32]. This paradoxical finding is consistent with a large body of previous studies conducted before the pandemic that found NHAAs had lower mental distress compared to NHWs and NHAs [33–35].

### The associations between SDOH and psychological distress

The present study advances understandings of associations between SDOH and psychological distress during the COVID-19 pandemic. Across the five domains of SDOH, we found that unemployment, food insecurity, housing instability, high educational attainment, usual source of health care, delayed medical care, and low neighborhood social cohesion and safety were associated with high levels of psychological distress in at least one racial/ethnic group.

Being employed was related to lower likelihood of moderate/severe psychological distress among NHWs, Hispanics, and NHAAs. Involuntary job loss can adversely impact mental health through mediating factors including financial strain as well as reduced personal control [36]. The COVID-19 pandemic resulted in a drastic increase in U.S. unemployment rates, from around 4% to 14% in March 2020 [37], when the data used in this study were collected. The negative relationship between employment and psychological distress was also documented by recent studies during the pandemic [38–40]. While Matthews et al.’s study (2021) found that job loss had the greatest effect on psychological distress in Blacks and Asians, the current study did not find this relationship among NHAs. One possible reason is that Matthews et al. (2021) analyzed K6 as a continuous variable. We administrated weighted multiple linear regression analysis using K6 score as a continuous variable and also found the negative relationship between employment and psychological distress among NHAs.

Food security was negatively related to psychological distress levels, which indicates food security was associated with the decreased psychological distress among NHWs and Hispanics. This finding is consistent with previous research [41]. Previous studies indicate that food insecurity is related to increased likelihood of mental distress [42, 43], which may be explained by chronic stress associated with striving to meet basic necessities; alternatively, low intake levels of essential nutrients may harm psychological functioning when enduring food insecurity [44, 45]. Hispanics and NHAAs faced the greatest food insecurity in this study, yet we found no significant associations between food insecurity and psychological distress among NHAA group, which was inconsistent with a pre-pandemic study using CHIS 2009–2012 [46]. These mixed findings call for post-pandemic examinations on the association between food security and psychological distress among NHAAs.

Notably, we found that NHW homeowners experienced a low likelihood of moderate/severe psychological distress. A previous study argued that the ontological security provided by owning a home might be the path through which home ownership impacts psychological outcome [47]. While the association between home ownership and better mental health has been established in previous studies [48–50], future research might focus on such associations among different racial/ethnic groups.

Our study found that NHAAs with bachelor’s degree or above were likely to report moderate/severe distress, though such relationship was not found in other groups. However, two previous studies used the same dataset of U.S. NHAAs and demonstrated the opposite finding, indicating that higher educational levels are related to lower levels of psychological distress [51, 52]. Recent studies conducted in China and Italy during the pandemic reported consistent findings with the current study [53, 54]. Qiu and colleagues (2020) suggested that a higher likelihood of moderate/severe psychological distress for participants with higher educational levels might be explained by their higher awareness of their health. However, such explanations fail to explain the racial/ethnic difference in current study. The mixed findings regarding the relationship between psychological distress and educational level demands further research, especially within research comparing different races/ethnicities.

Regarding health care access and quality, we found that having a usual source of health care (except emergency care) was associated with low odds of having moderate/severe psychological distress among NHAAs. Moreover, having experienced delayed medical care increased the likelihood of moderate/severe psychological distress among all groups, which is consistent with previous studies indicating negative relationships between delayed medical care and mental health outcomes [40, 55]. With an increasing number of individuals experiencing delayed medical care [56, 57], the COVID-19 pandemic can lead to increased morbidity and mortality that is not directly caused by viral infection [58–60]. In our study, NHAAs showed the strongest association between having delayed medical care and psychological distress across all four groups. Racial/ethnic differences of the relationship between health care access/quality (i.e., having a usual source of health care and having delayed medical care) and psychological distress might be explained by higher numbers of existing medical conditions among NHAAs. As the current study suggests, NHAAs showed the highest risk of severe illness from COVID-19, which included medical conditions such as asthma, diabetes, high blood pressure, heart disease, and obesity [26].

Non-Hispanic Whites who reported higher levels of neighborhood social cohesion were less likely to report moderate/severe psychological distress. Although Hispanic and NHAA groups had significantly lower levels of neighborhood social cohesion compared to NHWs, we found no significant relationships between neighborhood social cohesion and psychological distress among those two groups, nor was this association noted among NHAs. The relationship between neighborhood social cohesion and mental health remains inconclusive in previous studies, especially among racial/ethnic minorities. For example, some studies suggest that neighborhood social cohesion can be a positive factor associated with better mental health [61–65]. However, several earlier studies found no significant relationship between neighborhood social cohesion and mental health among African Americans [66], Hispanic Americans [67], and Asian Americans [68]. Notably, studies found significant relationships between neighborhood social cohesion and health outcomes among NHWs in the U.S., but this relationship was non-significant among other racial/ethnic groups [69, 70]. Future research should seek additional clarity regarding the nuanced distinctions across race/ethnicity as related to neighborhood social cohesion and mental health. Lastly, better neighborhood safety was associated with lower odds of moderate/severe psychological distress among all groups, which was reported by previous studies [66, 70–72]. Physical activity was reported as a mediator between neighborhood safety and mental health outcome, indicating that neighborhood safety concerns are negatively associated with physical activity, thus negatively related to mental health [71]. With social distancing recommendations limiting indoor exercise opportunities during the pandemic, it is unsurprising that lower neighborhood safety is highly associated with lack of physical activity, when considering that walking in the neighborhood is one of the few options for engaging in physical activity during the pandemic [73].

### Limitations

The findings of this study should be interpreted with caution due to several limitations. First, this study is not able to determine causal relationships between SDOH and psychological distress among participants due to the use of a cross-sectional data. Additionally, the self-reported nature of these survey data may pose limitations. For example, this study used a brief screening instrument (K6) to measure psychological distress of participants, which cannot determine the clinical outcomes among participants. Moreover, the current study is only able to examine individual-level SDOH. Psychological distress among participants might be impacted by multi-level factors [74]. Similarly, this study might not cover all SDOH that are associated with psychological distress, such as discrimination [75], health literacy [76], and social support [77]—measures which were not captured through these public-use data. Lastly, the study lacks generalizability to other geographic regions of the U.S. since the data were collected in California.

### Implications for public health practice and policy

Despite noted limitations, this study has implications for public health practice and policy. First, these findings highlight racial/ethnic disparities regarding psychological distress level and SDOH among four racial/ethnic groups. In combination with findings from previous studies, our study suggests that Hispanic adults are facing more adverse SDOH and are disproportionately impacted by the pandemic. Moreover, some social determinants were significantly associated with psychological distress levels among Hispanic adults, who presented with the highest rates of psychological distress among the four racial/ethnic groups in this study.

Although the U.S. implemented temporary support such as Employment Impact Payment Actions (i.e., coronavirus stimulus check), the CARES Act, and the families First Coronavirus Response Act across the nation, actions are still needed to address and dismantle the structural and cultural barriers to achieve economic stability during the COVID-19 pandemic. Regarding food insecurity, participants might face barriers, such as difficulties enrolling in Supplemental Nutrition Assistance Program (SNAP), problems accessing food banks during shutdowns, or securing transportation to food shopping [78]. Flexible enrollment and certification requirements for SNAP and outreach to the communities at higher risk of food insecurity are warranted to ensure particular racial/ethnic groups are not further marginalized [78]. Considering many individuals’ dependence on neighborhoods for outdoor activity in compliance with social distancing rules, the importance of perceived neighborhood safety might be heightened during the pandemic. Measures (e.g., improved outdoor lighting and maintenance) and support services (e.g., policing and public transportation) for neighborhoods perceived to be less safe should be considered [79].

Moreover, the strong relationship between delayed medical care and psychological distress among all groups highlighted the collateral damage experienced during the COVID-19 pandemic. Previous studies have demonstrated a drastic increase of delayed medical care among U.S. adults [56, 57]. With use of digital communication technologies, telehealth can (in many cases) deliver long-distance clinical health care, patient and provider education, health information and health administration services [80, 81]. Provider–patient communication through telehealth services can address many patients’ concerns regarding medical care. Tele-mental health services were also suggested by a previous study addressing mental health symptoms during the pandemic [82]. Additionally, health care accessibility and quality were suggested as important factors associated with psychological distress levels among NHAAs (McGuire & Miranda, 2008). Expanding health care accessibility, improving health care quality, and providing telehealth services to manage new and ongoing medical conditions in African American communities could help relieve psychological distress.

## Conclusion

This study highlighted different racial/ethnic groups are impacted by different SDOH at different level during the pandemic. The SDOH that are related to psychological distress also vary across different groups. Interventions aiming to reduce psychological distress among all groups should not be “one size fits all”. Our study suggests that Hispanic adults are facing more adverse SDOH and are disproportionately impacted by the pandemic. Public health practices and policies need to highlight SDOH that are associated with different racial/ethnic groups and develop tailored programs to reduce psychological distress.

## Data Availability

The dataset generated and/or analyzed during the current study is publicly available on CHIS website:https://healthpolicy.ucla.edu/chis/analyze/Pages/default.aspx.
